# The impact of virtual reality on curiosity, joy, and engagement

**DOI:** 10.3389/fpsyg.2026.1809238

**Published:** 2026-04-13

**Authors:** Minshu Zhang, BinBin Li

**Affiliations:** 1 College of Commerce and Tourism, Hunan Vocational College for Nationalities, Yueyang, China; 2 Faculty of Education, Southwest University, Chongqing, China

**Keywords:** curiosity, engagement, impact, joy, virtual reality

## Abstract

Since the introduction of virtual reality (VR) into the field of education, research on VR and education has gradually increased. However, most studies have primarily focused on the impact of VR on academic performance, with few examining its effects on curiosity, joy, and engagement. Even among the existing studies on VR and curiosity, joy, and engagement, few have examined the relationships among these three factors within a VR context. This raises questions regarding the positive and negative impacts of VR on curiosity, joy, and engagement in educational settings, as well as the relationships among these factors as elicited by VR. To address these questions, this article employs a narrative literature review method, analyzing 30 articles related to VR and curiosity, joy, and engagement. The findings revealed that VR-based learning environments hold greater potential for fostering curiosity, joy, and engagement compared to traditional teaching methods, as they provide immersive experiences and interactivity that encourage exploration and engagement. At the same time, joy mediates the relationship between VR and curiosity and engagement, stimulating students’ curiosity and leading to more active engagement. However, VR faces challenges such as insufficient depth of content interaction, lack of design complexity, high technical barriers, and inadequate teacher training and support systems, which may negatively impact its effects on curiosity, joy, and engagement. Therefore, when using VR in teaching, educators are encouraged to treat VR technology as a supplementary teaching tool rather than relying on it to fulfill all instructional tasks.

## 1 Introduction

Since the introduction of VR in education, research on VR and teaching has grown significantly, driving the development of VR in educational settings. VR primarily generated visual effects through head-mounted displays (HMDs) to enable interaction with virtual worlds, offering presence, interactivity, and immersion (Lee et al., 2017). In recent years, some authors have explored the impact of VR on presence and academic performance (Han et al., 2022; Lin et al., 2024; Shin, 2018). Others have examined the characteristics, advantages, and limitations of VR (Checa and Bustillo, 2020; Hamad and Jia, 2022; Tusher et al., 2024). However, despite the growing body of literature on VR, few studies have discussed its impact on curiosity, joy, and engagement. Curiosity was divided into transient state/task curiosity (context/task-specific) and trait curiosity, a relentless desire to learn something new (Kaczmarek et al., 2024). For the purposes of this study, curiosity refers to an individual’s desire to seek knowledge. The broaden-and-build theory, proposed by Fredrickson (2018), is based on the assumption that positive emotions, such as joy, one of the most important emotions alongside love and happiness, generate long-lasting personal resources, making people more resilient, enhancing their wellbeing, and improving their physical and mental health. In this study, joy specifically refers to the immediate, positive, and pleasurable feelings that arise during the learning process, often associated with the concept of flow. Engagement involved cognitive, behavioral, and affective aspects. Cognitive engagement entailed paying attention and understanding in depth; behavioral engagement was more observable; and affective engagement was associated with emotional reactions, such as motivation (Fredricks et al., 2004). This article discusses the three dimensions of engagement: cognitive, behavioral, and affective.

Although these specific variables have distinct underlying meanings, they are interconnected in a dynamic way. In immersive learning, joy serves as an emotional mediator that stimulates students’ curiosity and encourages their active engagement in learning. This article specifically selects curiosity, joy, and engagement—rather than other common emotional variables such as motivation, interest, or enjoyment—as the primary analytical categories because these capture a comprehensive learning mechanism. This is crucial for a more nuanced understanding of how VR environments influence students’ learning processes and outcomes. Furthermore, while theoretical relationships exist between these concepts and the learning process, few studies have examined them in an integrated manner. For example, in Cabrera-Duffaut et al.’s (2024) study, the search terms focused on VR-related concepts such as “learning,” “skills,” and “competence” (p. 4), rather than curiosity, joy, and engagement in VR. Similarly, the study by Kamińska et al. (2019) focused on the functions and applications of VR in the teaching process but did not examine the impact of VR on curiosity, joy, and engagement. A potential consequence of this lack of research in the field is that teachers may underestimate the impact on students’ overall learning outcomes, which in turn could affect teaching effectiveness.

In addition, researchers have recently begun to examine the impact of VR on curiosity, joy, and engagement (Cheng, 2023; Fang et al., 2021; Schutte, 2020). For example, Cheng (2023) recruited students from an immersive VR university to participate in an immersive virtual reality (IVR) experiment to explore the impact of VR on students’ epistemic curiosity, situational interest, and learning engagement. The results indicated that VR can benefit student learning in terms of motivation and engagement. Similarly, Schutte (2020) designed an interactive VR experience group and a control group to investigate the impact of VR on interest, joy, vitality, and flow. The results indicated that VR has a beneficial effect on these factors. These findings demonstrate the positive value of VR in fostering curiosity, joy, and engagement. However, Tu and Lee (2025) found that while VR may yield beneficial outcomes for curiosity, joy, and engagement, its effectiveness in enhancing these aspects might be limited when VR designs lack challenge and fail to satisfy users’ curiosity. These differing perspectives reflect variations in the understanding and effectiveness of VR across different educational settings. Therefore, further research into the impact of VR on curiosity, joy, and engagement is necessary. To achieve this goal, a good starting point is to conduct a systematic review of the literature in this field, exploring the impact of VR in education on curiosity, joy, and engagement.

The central research questions of this study are: What is the impact of VR on curiosity, joy, and engagement in educational settings? And what are the relationships among curiosity, joy, and engagement induced by VR? By addressing these research questions, we can clarify the positive and negative impact of VR and the underlying mechanisms linking these factors, thereby helping educators enhance their teaching capabilities and digital literacy, and providing researchers with insights into this emerging field and directions for future research. Therefore, to explore this topic in greater depth, this article is divided into four sections. First, the data collection methods used in this study will be reported. Second, the results of the research questions will be presented and discussed to reveal the impact of VR in education on curiosity, joy, and engagement, as well as the relationships among these factors. Finally, the conclusions will be summarized, and the limitations of this study and directions for future research will be identified, providing effective pathways for future studies.

## 2 Method

### 2.1 Search strategy

**Figure 1:**
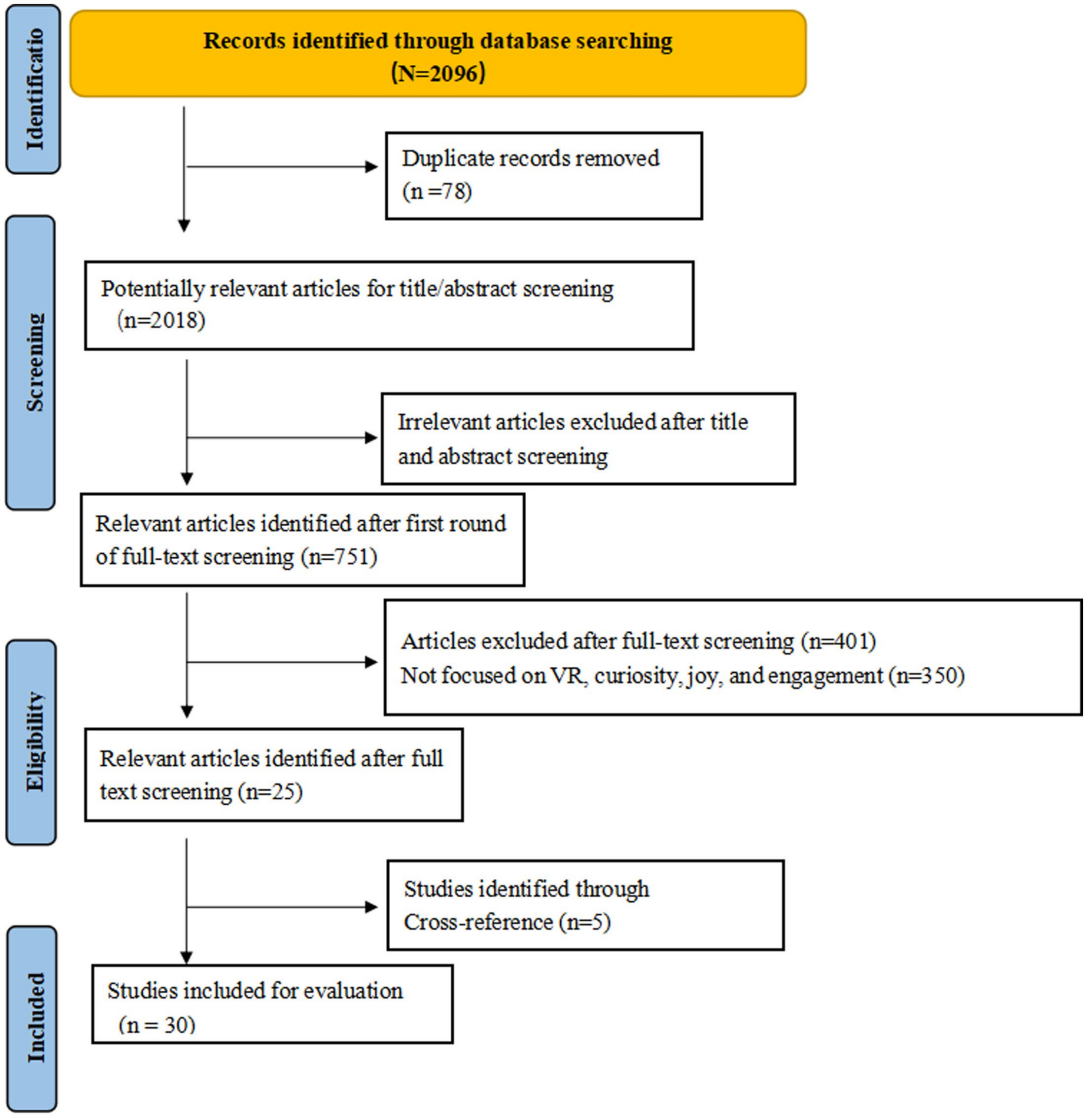
Systematic search strategy.

This study adhered to the relevant criteria outlined in the guidelines for narrative reviews (Green et al., 2006). Studies were included primarily based on the clarity of their research objectives, the rigor of their methodology, and their relevance to the study’s purpose. To enhance the transparency of the literature search and screening process, we employed a structured workflow and utilized the Preferred Reporting Items for Systematic Review and Meta-Analyses (PRISMA) (Moher et al., 2009) flowchart format to visually illustrate the key steps in literature identification and screening. The search strategy used in this review was based on (See Figure 1).

A narrative review method was employed in this study. Baumeister and Leary (1997) and Green et al. (2006) noted that narrative reviews can identify and synthesize relevant research on specific topics and/or research questions. This article aims to compile literature on the relationship between VR and curiosity, joy, and engagement. Therefore, this article summarizes previous reviews as follows. First, information was retrieved from the SAGE, Springer, ScienceDirect, JSTOR, and Taylor Francis databases using the search terms: “virtual reality” AND “curiosity” AND “joy” AND “engagement.” The search was limited to English-language articles published between 2015 and 2025. The initial search used all combinations of the aforementioned keywords and, following a preliminary screening, yielded 2,018 potentially relevant articles (SAGE: 223, Springer: 831, ScienceDirect: 217, JSTOR: 99, Taylor Francis: 648).

In the second phase, secondary keywords such as “education” AND “impact” AND “teaching” were added, reducing the number of articles to 751 (SAGE: 80, Springer: 345, ScienceDirect: 74, JSTOR: 71, Taylor Francis: 181). Of these, 1,267 did not meet the criteria and were excluded. These articles were excluded because their target audience was technology developers. In the final stage, an additional 726 articles were excluded because they were duplicates or did not fully explore the research topic in the full text. Furthermore, by manually searching the references of articles related to the topic, additional articles were identified and evaluated, of which 5 met the eligibility criteria. Consequently, 5 articles were added to the previously identified 25. Finally, the full texts of these articles were reviewed to determine whether their work aligned with the focus of this review. Thirty articles passed the final review, covering samples related to VR and curiosity, joy, and engagement, and were included in the analysis.

### 2.2 Inclusion and exclusion criteria

Based on the work of Woods et al. (2011), inclusion and exclusion criteria were established. Following Ezzamel and Bond (2016), these criteria were designed to maintain focus on the research question and effectively exclude studies that extend the scope of the review beyond the research question. To ensure the quality of the literature, we selected only peer-reviewed English-language articles published within the last decade. Articles written in languages other than English, as well as those published outside the specified time and language parameters, were excluded. To ensure the comprehensiveness, representativeness, and reproducibility of the search, we included five databases: SAGE, Springer, ScienceDirect, JSTOR and Taylor Francis. These databases were selected because they provide high-quality academic articles in fields such as education, psychology, and technology, thereby ensuring broad coverage, disciplinary relevance, and high-quality indexing. Other databases, such as Scopus and PsycINFO, were excluded because they contain relatively few studies relevant to this topic and there was partial overlap with the articles included in the databases selected for this study. Following the principles of theoretical saturation and relevance, we included 30 studies to support the answers to the research questions. The sample population encompassed studies conducted in elementary, secondary, and university settings. Moreover, articles employing qualitative, quantitative, and mixed-methods approaches were included to synthesize the available literature in this field.

During the literature search phase, in the first stage, we used a Boolean search with the core keywords “virtual reality” AND “curiosity” AND “joy” AND “engagement” to ensure that the initial results focused on the core concepts. In the second stage, to further narrow the scope of the study and examine the impact of VR on curiosity, joy, and engagement, as well as the relationships among these factors, we added secondary keywords such as “education” AND “impact” AND “teaching” to the above search terms. Regarding inclusion criteria, we did not include studies merely because these terms appeared in the keywords or abstract; rather, we required that these concepts be thoroughly discussed in the full text. Studies in which relevant terms appeared only in the keywords or abstract without systematic analysis in the full text were excluded. The literature screening process consists of two stages: “initial screening of titles and abstracts” and “detailed screening of full texts.” First, based on the titles and abstracts, we determine whether the studies involve VR applications in educational settings, with a focus on curiosity, joy, and engagement. Second, we read the full texts of potentially eligible studies to assess whether they meet the predefined inclusion criteria, thereby avoiding the inclusion of studies that only indirectly or superficially address these concepts.

### 2.3 Data extraction and analysis

The records were identified and processed using PICOS categories and other relevant variables. The PICOS framework was applied by adding study design to the original PICO elements (Participants, Intervention, Comparison, Outcomes) (Cooke et al., 2012; Methley et al., 2014; Tawfik et al., 2019). Specifically, PICOS was used to structure the research question, develop the coding tool, and guide the narrative literature review, including the literature search, study comparison, and synthesis of evidence (Cooke et al., 2012; Methley et al., 2014; Tawfik et al., 2019). Within this article, PICOS enables analysis of (a) country, (b) participants, (c) study design, (d) intervention, (e) results, and (f) limitations. Table 1 summarizes the identified studies.

**Table 1:** Articles included in the systematic review.

Author and country	Participants	Study design	Intervention	Outcome	Limitations
Alam and Mohanty (2023), India	A study involving 122 fifth-grade students in Hyderabad, India—61 boys and 61 girls—with an average age of 10.11 years.	Mixed-method research method	The study examined the impact of VR on students’ emotional experiences, particularly in terms of joy.	VR had a positive impact on students’ sense of joy.	Most participants had no prior exposure to VR-based education; the study results may have been positively influenced by the novelty and diffusion effects.
Bluman et al. (2023), Israel	124 participants aged 19–62 at a bar in central Tel Aviv	Quantitative research method	Explored the relationship between positive emotions and engagement in a VR environment.	Positive emotions in VR environments can enhance engagement.	Effectiveness across different populations is not yet validated.
Barpi et al. (2023), Italy	Two universities	Qualitative research method	Emphasizing the mediating role of joy stimulated curiosity and motivated further learning engagement.	The positive and negative impacts of emotions on shaping students’ learning, engagement, and curiosity in traditional and virtual learning environments.	The data are from China only and it may be difficult to generalize the results to other regions. Sample size has not been specified.
Brylska et al. (2024), Poland	75 people, including 42 women and 33 men, aged 19–24.	Mixed-method research method	The study compared the effects of VR and traditional classroom instruction on students’ knowledge retention and emotional responses.	VR environments are more conducive to knowledge retention and evoke positive joy than traditional classroom instruction, but they may also lead to excessive cognitive load.	The grouping method may have limited the identification of additional subgroups. It failed to fully reveal differences between groups and individual characteristics.
Cheng et al. (2023), China	90 elementary school students.	Quantitative research method	The relationship between cognitive curiosity, situational interest, and attitude learning is confirmed. Joy during VR learning is identified as a mediating variable.	Students’ joy mediated the relationship between curiosity and learning engagement.	Participants possessed prior knowledge of the learning topic, so the findings may not adequately represent the affective-oriented perceptions of younger learners lacking scientific knowledge when engaging with interactive VR learning.
Chen et al. (2025), China	Second- and third-year students in the School of Design at a university in China. There are 208 male students and 317 female students.	Mixed-method research method	This study examines the positive and negative impacts of VR immersion, presence, joy, engagement, enjoyment, and flow.	Compared to traditional teaching approaches, VR’s immersive experience and sense of presence can enhance students’ joy, engagement, enjoyment, and flow.	The research is limited to a single school in China, making it difficult to generalize the findings to broader national contexts or educational levels.
Dubovi (2022), Israel	61 first-year nursing students at Israeli universities.	Mixed-method research method	Investigated the mediating role of emotions, particularly joy, in VR.	VR induced higher student engagement, greater pleasure, and prompted greater psychological effort.	The study selected only variables most strongly and consistently correlated with outcome variables for subsequent regression analysis, potentially obscuring important statistical interactions.
Dai and Wang (2024), China	631 participants aged 18–46 from universities across China were involved. Among them, 143 were male and 488 were female.	Quantitative research method	Explored the interrelationships among various factors such as anxiety, boredom, enjoyment, and student engagement in VR.	It revealed a positive correlation between joy and learning engagement.	The study’s focus solely on Chinese English learners’ joy and engagement in technology-based English-taught classrooms may lead to oversimplification or overlook nuanced differences.
Georgiou et al. (2021), Cyprus	109 high school students (grades 10–12) from eight fully equipped physics classrooms across five schools. The group consisted of 51 female and 58 male students, with an average age of 15.76 years.	Mixed-method research	The study assessed students’ perceptions of the immersive learning experience.	VR can foster students’ enthusiasm for learning.	The study did not include a broader range of immersive VR simulations or students from different age groups and subject areas. The schools participating in the study were carefully selected, which limits the generalizability of the findings.
Huang et al. (2023), USA	A city in a state in the southeastern United States, featuring a natural park and a nearby university. 120 participants (67 women, 53 men).	Qualitative research method	The study investigated the effects of IVR on science learning, enjoyment, and motivation, and compared these outcomes with those in real-world settings.	VR had positive effects on science learning, enjoyment, and motivation, and can serve as an alternative to in-person, nature-based experiences.	The study did not examine how different levels of interaction in VR design affect the expected learning and motivational outcomes.
Hsu (2024), Taiwan, China	230 participants: 128 women and 102 men.	Quantitative research method	This study examined the impact of VR immersion on engagement, curiosity, and joy among English as a Foreign Language (EFL) learners.	VR immersion has a positive impact on EFL learners’ engagement, curiosity, and enjoyment in language learning.	The study focused solely on foreign language disciplines. It did not include a more culturally diverse sample of participants. No post-test or longitudinal studies were conducted.
Jin and Zhong (2025), China	67 students from two ninth-grade physics classes at a middle school in Wuhan, Hubei Province.	Quantitative research method	This study examined the effects of learning in immersive virtual environments on students’ joy and engagement.	Immersive virtual learning enhances joy, engagement, and self-efficacy.	Self-report questionnaires may introduce subjective bias. The relatively short intervention period limited the assessment of long-term learning outcomes.
Kurniawati et al. (2019), Indonesia	Two schools, 8 students from SLBN Keleyan; 5 students from Sekolah Khusus Bina Mandiri. Age range is between 6 and 20 years old.	Quantitative research method	This study presents the first research on VR games for students with special educational needs (SEN), exploring their impact on stimulating curiosity, interest, and engagement among learners.	VR games stimulate children’s curiosity, joy, and behavioral engagement in the classroom.	Findings and data originate from laboratory settings, where conditions may differ from real-world scenarios.
Lee et al. (2022), Taiwan, China	74 sixth-grade students from an after-school tutoring program at an elementary school in central Taiwan. There were 32 girls and 42 boys.	Quantitative research method	This study examined the effects of VR on students’ cognitive curiosity and positive joy, comparing two groups of students.	The effects of VR on emotional factors such as cognitive curiosity and positive joy yielded opposite results in certain aspects between the two groups.	The sample size was small. The self-administered questionnaire used to assess students’ joy made it difficult to capture the instantaneous changes in joy across different IVR scenarios.
Laine et al. (2023), Finland	Three teachers and 59 students (ages 10–12) from two elementary schools in Finland	Qualitative research method	To explore the learning experiences of elementary school students using VR.	Interactive virtual reality (IVR) has both positive and negative effects on students’ learning motivation, desire to learn, flow experiences, and engagement.	The sample size is limited.
Makransky and Petersen (2021), Denmark	Two researchers.	Quantitative research method	The study examined the emotional and cognitive factors of IVR on learning outcomes such as joy, curiosity, and engagement, with a particular focus on joy.	IVR can enhance cognitive and affective factors, but it may also present challenges in these areas.	Other relevant variables were not included. The study did not investigate the impact of external factors on the different variables in the model and their relationships.
Makransky and Mayer (2022), Nationwide	102 secondary school students aged 13–16 (mean age 14.14) from four public schools in different regions of a European country. There were 39 male students and 63 female students.	Qualitative research method	The study investigated the effects of IVR on science learning, enjoyment, and motivation, and compared these outcomes with those in real-world settings.	Virtual field trips using head-mounted displays (HMDs) outperformed similar virtual field trips presented in two-dimensional video format in terms of immersion, enjoyment, and motivation.	The sample size was small. Additional factors influencing the immersive learning process were not included.
Muthmainnah et al. (2023), Indonesia	350 undergraduate students in English language programs.	Quantitative research method	The study examined the impact of VR on Indonesian English learners’ creativity, engagement, motivation, and enjoyment.	VR can enhance Indonesian English learners’ creativity, creative writing skills, academic performance, engagement, motivation, and enjoyment.	The sample is limited to a single country, which restricts the generalizability of the conclusions.
Marchioro et al. (2024), Mexico	30 boys and 30 girls, aged 8–12.	Case study	This study highlights VR’s potential to stimulate and engage children’s interest and engagement. It also explores the future prospects of VR becoming a significant tool in education.	VR technology can adapt to the learning styles and needs of diverse student groups, promote emotional recovery, and counteract low learning motivation.	The research sample is limited to Mexican children, making it difficult to generalize the findings to other countries and populations.
Merchán et al. (2025), Spain	135 third-year students enrolled in the social science teaching methodology elective.	Mixed-method research method	Explores how to enhance teaching effectiveness among early childhood teacher trainees. Evaluates the efficacy of VR in fostering emotional engagement and engagement.	Students demonstrated improvements in knowledge, technical skills, positive affect, and engagement.	Convenience sampling limits the generalizability of findings.
Parong and Mayer (2021), USA	Eighty undergraduate students from a university in Southern California, aged 17–23 (mean age 19.28), including 19 males and 61 females.	Quantitative research method	The study compared the effects of IVR and traditional media on student learning outcomes, particularly in terms of affective and cognitive mechanisms.	IVR may generate excessive positive joy, which could distract students and consequently affect subsequent learning outcomes.	The practice tests in the experiment were conducted orally, which did not align with the final written exam format. The type of media may have been confused with the different physical interaction methods used in the two classes.
Rogers et al. (2022), Australia	52 undergraduate psychology students from Edith Cowan University, comprising 10 males and 42 females, with an average age of 31.73 years.	Qualitative research method	Compared the emotional impact of social interactions in VR versus face-to-face interactions on students.	Indicated that VR environments may have limited potential for enhancing students’ joy. Behavioral authenticity may be a more significant factor influencing the enjoyment of conversations in VR.	Sample bias exists, as the study primarily involved socially active women around 30 years old with limited experience in VR.
Ritter et al. (2024), US	Three leaders in the education technology industry.	Qualitative research method	Explore the positive impact of VR on joy, curiosity, and engagement.	VR can spark joy, curiosity, and active engagement, enhancing student learning outcomes.	Self-reported data from experts may be subject to common biases.
Sagnier et al. (2020), France	89 undergraduate psychology students and master’s students in engineering. 45 women and 44 men, aged 18–29.	Quantitative research method	The study examined the effects of VR on students’ joy and creativity.	VR can enhance students’ joy and creativity, but it may cause motion sickness.	Mean-variance extraction values for some subscales were relatively low, and the cutoff scores for some subscales were low. There were differences in the questionnaire structure between the French version and the original version.
Scorolli et al. (2023), Italy	Live Tango Concert at the Pavarotti-Freni Municipal Theater with 70 participants.	Qualitative research method	Exploring the impact of VR technology on enhancing curiosity, emotional engagement, aesthetic appreciation, and engagement among a specific demographic (i.e., young students).	VR can enhance curiosity, emotional engagement, aesthetic appreciation, and engagement among young students.	Less than half of the sample had prior virtual experience before the experiment; the novelty of using virtual devices for the first time may have introduced bias in the results.
Tafazoli (2024), Iran	34 in-service Persian language teachers from two Iranian universities.	Case study	Explores the potential for Iranian teachers to use VR in cultural instruction.	Following the workshop, teachers’ perceptions regarding VR’s application in cultural instruction underwent a positive shift.	The lack of participants with prior exposure to VR in educational settings may limit the generalizability of the findings.
Wolf et al. (2025), Austria	Thirty participants from Graz University of Technology, with an average age of 27.1 years.	Quantitative research method	The study evaluated the effects of VR on students’ innovative abilities, learning motivation, learning engagement, and enjoyment.	The VR Learning Factory enhanced learning motivation and creativity while reducing cognitive load.	The study results apply only to short-term participation and are limited by the inability to control for baseline factors among learners. The study was conducted at a single institution with a small sample size.
Ye et al. (2022), USA	463 primary and secondary school students (grades 9–10; 284 males, 179 females)	Mixed-method research	This study examined the determinants influencing learners’ willingness to continue using VR for learning in formal classroom settings.	Factors related to well-being, such as curiosity and joy, have a positive impact on students’ willingness to continue using VR for learning.	Other key factors, such as accessibility, teacher adaptability and preparedness, and access to resources, were not addressed.
Zainuddin et al. (2020), Indonesia	94 students aged 15–16 from three different science classes in Indonesian secondary schools.	Mixed-method research method	This study examines the impact of game-based VR platforms on engagement, joy, curiosity, and emotional responses, and explores the relationships among these factors.	VR can enhance engagement, joy, and curiosity.	Small sample size. Psychological data collected via questionnaires and interviews may be subject to various biases.
Zwoliński and Kamińska (2024), USA	254 students aged 18–25 who had experience with VR and were familiar with the theory of flow.	Qualitative research method	The study examined the effects of VR on learners’ joy, curiosity, and engagement.	VR can enhance learners’ joy, curiosity, and engagement.	Self-report questionnaires may introduce subjective bias.

## 3 Results and discussion

### 3.1 What are the positive and negative impacts of VR on curiosity, joy, and engagement?

#### 3.1.1 Curiosity

In their study on the impact of VR technology on stimulating curiosity, Cheng et al. (2023) proposed that VR technology could provide experiences that stimulate curiosity, which stems from a desire to learn and understand new information and experiences. Scorolli et al. (2023) also found that after engaging with VR experiences during visits to museum or art gallery exhibitions, students reported that VR stimulated their curiosity to experience and learn new things. Similarly, Kurniawati et al. (2019) found that VR games typically feature strong visual effects and convenience; by providing immersive 3D environments, they could stimulate K-12 students with Special Educational Needs (SEN) to learn through gameplay and support their free exploration in the classroom. Therefore, VR provides students from diverse groups with opportunities to engage with, explore, and interact with new information; these opportunities may spark curiosity, which in turn drives students to actively seek out new knowledge, information, experiences, and the act of learning itself. Students with a strong sense of curiosity tended to feel more uneasy about knowledge gaps, which further strengthens their motivation to expand their knowledge or skills and seek challenging and complex tasks or solutions to engage in meaningful learning activities (Scorolli et al., 2023).

However, some researchers found inconsistent results. A study by Ritter et al. (2024) found that VR might be negatively correlated with students’ curiosity; students with high curiosity were less inclined to seek novel and challenging scenarios in VR. This may be because students with high levels of curiosity perceive current VR applications as insufficiently challenging or overly restrictive in VR environments, leading them to seek out fewer novel or complex tasks and thereby reducing their intrinsic motivation to satisfy their curiosity through challenging tasks. Lee et al. (2022) also observed that under different instructional designs, the effects of VR on cognitive curiosity and the experience of joy can even yield opposite results. Furthermore, the study by Ritter et al. (2024) further pointed out that current VR instruction primarily relies on simple interactions and lacks deep-level interactive design, which might limit students’ enjoyment of active exploration and the cultivation of curiosity. This aligns with the findings of Chen et al. (2025), who found that VR platforms tended to be one-way observation experiences with insufficient interactivity and personalized instructional support, making it difficult to stimulate students’ motivation to actively seek knowledge. These findings highlight both the positive and negative impacts of VR on curiosity.

The differing results of these studies stem largely from variations in methodology and design. In the study by Scorolli et al. (2023), less than half of the participants had prior experience with virtual reality; consequently, the findings might be influenced by the novelty effect, meaning that the stimulation of curiosity stemmed more from the novelty of the technology than from the educational value of VR. Similarly, while Kurniawati et al. (2019) demonstrated VR’s impact on stimulating curiosity, joy, and engagement among students with special educational needs (SEN), the study’s small sample size and the fact that the results and data were derived from a laboratory setting rather than a real classroom environment cast doubt on its ecological validity. More critically, studies by Ritter et al. (2024) and Chen et al. (2025) revealed that highly immersive VR environments differ from simple interactive VR environments in terms of interaction depth; one-way observational VR reduces the likelihood of learners engaging in active exploration, while highly immersive VR, by reducing perceived task risk and anxiety levels, might diminish the curiosity of learners who would otherwise prefer complex and high-risk tasks. Therefore, rather than simply viewing VR as a tool for stimulating curiosity, it is better understood as a contextual moderator, whose effectiveness depends largely on the interaction of multiple factors, including task design complexity, challenge level, learners’ prior experience, and individual traits.

#### 3.1.2 Joy

Brylska et al. (2024) found that VR-based scenarios might elicit stronger emotional responses compared to non-immersive environments, particularly with regard to the specific emotion of joy. For example, specific scenarios in VR settings—such as interacting with sculptures and exhibits in a virtual museum—could lead students to experience various emotions, including happiness, joy, and fear, with intensities far exceeding those in other scenarios (Merchán et al., 2025). Bluman et al. (2023) also found similar results, showing that affect-induction procedures (AIPs) in interactive VR produced higher levels of joy and reduced negative emotions compared to AIPs in non-interactive VR. Since positive emotions are often negatively correlated with negative emotions, the increase in joy may be associated with a reduction in negative emotions. Similarly, the study by Dai and Wang (2024) confirmed that technology-based learning environments are more effective than traditional teaching environments at eliciting positive feelings such as joy. In other words, the immersive nature of VR creates a sense of presence, enabling students to actively engage in emotional experiences and thereby trigger emotional arousal. These findings demonstrate the relationship between immersion, user experience, and joy responses in VR scenarios.

However, these findings are subject to certain limitations across different studies and may even have potential negative effects. Regarding design variations, while these studies have found that VR can induce joy to a significant extent, it remains unclear how VR experiences should be designed to be effective, or whether certain design decisions influence the intensity of the joy experienced. In fact, VR does not inherently induce joy; its effectiveness depends on the quality of interaction and the realism of the scenario. Laine et al. (2023) noted that the limited realism of VR visuals and movements could undermine students’ immersion and emotional connection; most people might prefer face-to-face interactions over those in virtual reality. Thus, while supportive social networks are closely linked to physical and mental health and wellbeing, it remains unclear whether this effect holds true in virtual environments.

Regarding individual baseline differences in joy, Dai and Wang (2024) noted that interactive VR experiences could enhance participants’ sense of joy only when their prior emotional state was neutral; under negative emotional conditions, however, the joy-regulating effects of interactive VR experiences were limited. Currently, most studies on VR and joy focus on learners with relatively neutral or positive initial joy states, which may overestimate the general applicability of joy among students with more diverse or negative emotions. To this end, Rogers et al. (2022) suggested that the impact of increased VR use on human negative emotions should also be considered, as research indicates that increased screen time was associated with adverse consequences such as reduced sleep, increased sedentary behavior, depression, attention problems, and negative emotions. It was worth noting that Makransky and Mayer (2022) also pointed out that the complex interfaces and functions of VR presented a learning barrier for teachers and students with lower digital literacy; the lack of systematic teacher training and technical support might hinder the full utilization of the platform, thereby generating emotions such as anger, confusion, and boredom. Studies by Alam and Mohanty (2023) and Hsu (2024) also acknowledged limitations in their findings due to the exclusion of additional factors and a lack of a broader, more diverse participant pool. Therefore, the role of VR in promoting joy is contingent upon certain preconditions rather than a universal mechanism. The impact of VR on joy depends not only on the technology itself but also on factors such as the instructional framework and support structures.

#### 3.1.3 Engagement

Studies by Huang et al. (2023), Lee et al. (2022), and Makransky and Petersen (2021) have all found that, compared to traditional teaching methods, students achieve higher academic performance, enjoy a more engaging classroom experience, and demonstrate greater engagement when learning in VR environments. In other words, immersive VR learning experiences help students gain a deeper understanding of complex and abstract concepts and enhance their cognitive engagement. Zainuddin et al. (2020) also revealed that diverse exploration opportunities within interactive VR gamified designs—such as points, badges, certificates, and leaderboards—could further promote learning engagement. Barpi et al. (2023) further noted that the benefit of incorporating virtual instruction into the educational process was that it provides a safe and supportive environment for students who were less active in real-world classrooms, allowing them to express themselves and thereby increasing behavioral engagement. Moreover, Muthmainnah et al. (2023) observed that VR supports multi-user role-playing and situational interactions, which could strengthen connections and foster a sense of closeness among students. In other words, students can create a virtual avatar in the VR space, engage in real-time voice and motion communication, participate in virtual gatherings, and make new friends without fear of judgment or embarrassment, thereby enhancing affective engagement.

Although VR offered potential benefits in terms of cognitive, behavioral, and affective engagement, it also has limitations, such as insufficient depth of content interaction, design challenges, and inadequate teacher training and support systems (Georgiou et al., 2021; Jin and Zhong, 2025; Zwoliński and Kamińska, 2024). Educators faced ambiguous guidelines when integrating virtual content into their curricula, often having to navigate complex technical hurdles and master operational procedures on their own (Wolf et al., 2025). This implies that, without pedagogical support and contextual guidance, student engagement in VR often remains at the sensory level and fails to translate into deep cognitive processing. Georgiou et al. (2021) and Parong and Mayer (2021) found, through comparisons of virtual conditions with varying degrees of immersion, that high immersion does not always yield greater learning engagement; excessive sensory stimulation might actually distract students, thereby affecting their sustained engagement. This finding challenges the linear assumption that higher immersion leads to higher engagement. Furthermore, Sagnier et al. (2020) also raised concerns about the potential occurrence of motion sickness in virtual social environments, which could affect students’ sense of security and platform acceptance, thereby hindering deep engagement. This concern is valid, as motion sickness can lead to adverse effects such as nausea and dizziness, which in turn impede widespread student engagement.

A comparison of these studies reveals that the positive impacts of VR depend to some extent on intervention design and experimental conditions, rather than on the technology itself. Methodological differences across studies also contribute to the inconsistency in their conclusions. On the one hand, most studies—such as those by Lee et al. (2022) and Sagnier et al. (2020), employ short-term participation and controlled scenarios, making it easier to capture the immediate engagement generated by VR but difficult to assess the sustainability of that engagement. On the other hand, some studies rely on self-reports or single behavioral indicators to measure engagement, such as the studies by Jin and Zhong (2025) and Zwoliński and Kamińska (2024), making it difficult to distinguish between superficial and deep engagement, which may lead to an overestimation of VR’s actual effects. At the same time, some studies treat engagement as a comprehensive concept rather than a multidimensional trait—such as the work by Alam and Mohanty (2023)—failing to clearly distinguish between cognitive, behavioral, and affective dimensions. These conceptual differences limit the comparability of findings across studies.

Overall, compared to non-VR environments, interactive VR experiences tend to elicit higher levels of curiosity, joy, and engagement. However, VR also faces widespread challenges, including insufficient depth of content interaction and design complexity, high technical barriers, and inadequate teacher training and support systems. These findings not only help teachers better understand the strengths and limitations of VR-based instruction but also lay the groundwork for further exploration of the relationship between VR and these three factors: curiosity, joy, and engagement.

### 3.2 What is the relationship between curiosity, joy, and engagement triggered by VR?

The study not only examined the impact of VR on three key variables—curiosity, joy, and engagement—but also explored the relationships among them in greater depth. Dubovi (2022) confirmed the mediating role of joy between curiosity and engagement. VR experiences often evoke a sense of joy, and this joy might serve as a process or pathway that connects or moderates the relationship between curiosity and engagement, thereby linking the two (Dubovi, 2022). Similarly, in the study by Cheng et al. (2023), the joy elicited by VR experiences links VR’s impact on curiosity and engagement. In other words, within these immersive environments, joy serves as a driving force that stimulates students’ curiosity and encourages them to actively engage with the learning content. When students experience joy regarding different concepts or topics, their curiosity is sparked, leading to deeper exploration and learning. Ye et al. (2022) proposed that positive emotions such as joy, encouraged students to actively explore and broaden their horizons, continuously accumulating resources through this process. Curiosity, in turn, may facilitate the expansion and accumulation of these resources, thereby creating a virtuous cycle of wellbeing and a positive lifestyle.

Tafazoli (2024) further found that this high level of joy, in turn, encouraged students to actively engage in the learning process, as they were more proactive in seeking new information, exploring new resources, and participating in discussions and activities. This suggests that if the joy elicited by VR experiences is fulfilled, it has the potential to enhance engagement and lead to better learning outcomes. A study by Marchioro et al. (2024) reached similar conclusions, finding that joy could directly and indirectly predict student engagement and had a positive impact on students’ learning behaviors and classroom participation. Consequently, students experiencing joy may participate more actively in learning and invest more effort in their learning explorations. Furthermore, the study by Ritter et al. (2024) further revealed a positive correlation between curiosity and engagement, with students exhibiting higher levels of curiosity being more inclined to engage more deeply in learning activities.

However, the study by Chen et al. (2025) identified inconsistent results, suggesting that curiosity might also lead to a decline in engagement, thereby hindering student engagement in art field trips. This highlights an area that warrants further exploration. This may be because curiosity stems from a desire to reduce uncertainty or anxiety regarding a specific topic; it may not inherently possess the capacity to foster sustained interest or deep engagement in learning activities. Therefore, in VR contexts, while curiosity may guide students to quickly identify correct objects or acquire new knowledge—thereby alleviating initial doubts—this does not necessarily translate into deeper engagement or long-term interest. Once immediate concerns are resolved, the motivation driven by curiosity weakens, and students may no longer feel compelled to further explore or delve deeper into related concepts. These studies not only highlight the importance of joy in fostering curiosity and engagement but also underscore the influence of curiosity on student engagement and learning outcomes. Consequently, the dynamic interplay between curiosity, joy, and engagement creates a synergistic impact that collectively shapes both teaching practices and student learning.

A comparison of different studies suggests that the conflicting results may stem from variations in how “joy” is defined across studies. Some studies regard it as a general pleasure or interest (e.g., Cheng et al., 2023); others regard it as a high-arousal emotion (e.g., Chen et al., 2025); and still others, such as Ye et al. (2022), adopted broader terms such as “positive affect,” “fun,” or “enjoyment.” This conceptual overlap may limit the generalizability of findings across studies and lead to overinterpretation of mediating effects. Furthermore, most studies exhibit some degree of bias or systematic limitations, undermining the robustness of their conclusions. The majority of studies focus on student populations with high levels of technological acceptance, which may amplify the positive joy effects of VR. Moreover, studies generally overlook the moderating impact of individual differences—such as prior experience and emotional baseline—on the mechanism pathways. Together, these factors mean that current evidence supporting the argument that joy may mediate the relationship between curiosity and engagement remains largely based on indirect inferences, lacking rigorous causal validation. These findings serve as a reminder from various angles that educators should carefully examine the role of technology in learning and teaching.

## 4 Conclusions and limitations

This article discusses both the beneficial and adverse effects of VR on student curiosity, their happiness, and interest in learning, and the correlations among these variables, uncovering the benefits and detriments of VR in teaching. Results indicate that VR is effective in cultivating the three factors, where joy moderates the VR relationships with curiosity and engagement. However, the three factors might decrease with non-challenging VR content to highly curious learners; combined with poor example interaction, design issues, high technical levels, and insufficient teacher training and support, VR might also have negative influences. The research provides information on future relevant studies.

Among the limitations, there is no distinction between the learners of the varying age/developmental stages because they might have different responses to VR, and therefore, future research can examine the effectiveness of VR in various levels of education. Meanwhile, only curiosity, joy, and engagement are investigated with disregard for other factors, such as empathy, that can influence teaching results, as they should be investigated in further research to gain more knowledge about the pedagogical potential of VR.
